# Widespread Genotype-Phenotype Correlations in Intellectual Disability

**DOI:** 10.3389/fpsyt.2018.00535

**Published:** 2018-10-29

**Authors:** Emily L. Casanova, Zachary Gerstner, Julia L. Sharp, Manuel F. Casanova, Frank Alex Feltus

**Affiliations:** ^1^Department of Biomedical Sciences, University of South Carolina School of Medicine at Greenville, Greenville, SC, United States; ^2^Department of Pediatrics, Greenville Health System, Greenville, SC, United States; ^3^Department of Genetics and Biochemistry, Clemson University, Clemson, SC, United States; ^4^Department of Statistics, Colorado State University, Fort Collins, CO, United States; ^5^Center for Human Genetics, Clemson University, Clemson, SC, United States; ^6^Biomedical Data Science and Informatics Program, Clemson University, Clemson, SC, United States

**Keywords:** autism spectrum disorder, epilepsy, craniofacial abnormalities, neurodegeneration, infantile proteopathy, genetic phenotype associations

## Abstract

**Background:** Linking genotype to phenotype is a major aim of genetics research, yet the underlying biochemical mechanisms of many complex conditions continue to remain elusive. Recent research provides evidence that relevant gene-phenotype associations are discoverable in the study of intellectual disability (ID). Here we expand on that work, identifying distinctive gene interaction modules with unique enrichment patterns reflective of associated clinical features in ID.

**Methods:** Two hundred twelve forms of monogenic ID were curated according to comorbidities with autism and epilepsy. These groups were further subdivided according to secondary clinical manifestations of complex vs. simple facial dysmorphia and neurodegenerative-like features due to their clinical prominence, modest symptom overlap, and probable etiological divergence. An aggregate gene interaction ID network for these phenotype subgroups was discovered via a public database of known gene interactions: protein-protein, genetic, and mRNA coexpression. Additional annotation resources (Gene Ontology, Human Phenotype Ontology, TRANSFAC/JASPAR, and KEGG/WikiPathways) were utilized to assess functional and phenotypic enrichment patterns within subgroups.

**Results:** Phenotypic analysis revealed high rates of complex facial dysmorphia in ID with comorbid autism. In contrast, neurodegenerative-like features were overrepresented in ID with epilepsy. Network analysis subsequently showed that gene groups divided according to clinical features of interest resulted in distinctive interaction clusters, with unique functional enrichments according to gene set.

**Conclusions:** These data suggest that specific comorbid and secondary clinical features in ID are predictive of underlying genotype. In summary, ID form unique clusters, which are comprised of individual conditions with remarkable genotypic and phenotypic overlap.

## Background

Phenomics is a new and emerging area of study, underlying the development of genotype-phenotype mapping and the identification of different disease interaction networks (1). Genotype-phenotype mapping involves the delineation of a relationship between the genetic constitution of an individual and an observable set of characteristics of interest. While the relationship between a phenotype and a given genotype is complex, in the case of rare disorders with high-penetrance mutations determining such a relationship becomes relatively easier, although may still require the aid of computational methods such as those employed in the following study.

Intellectual disability (ID) is a complex and highly heterogeneous group of disorders despite cognitive and behavioral overlap. Genotype-phenotype correlations have been reported within individual ID syndromes and across different mutations within a given gene, yet only recently have there been reports of more extensive and generalizable genotype-phenotype clusters comprising subsets of the condition.

For instance, Casanova et al. (2) produced a manually curated catalog of 650 ID-associated genes that were grouped according to the presence of syndromic ID, non-syndromic ID, and multisystemic disorders. In addition, conditions were further subdivided according to variations in severity and mutation penetrance. The team identified a variety of functional enrichment patterns associated with specific clinical manifestations, such as MAPK, growth factor, and DNA repair signaling in conditions exhibiting short stature and ectodermal anomalies; microcephaly and behavioral features associated with genes enriched for chromatin-related functions, which regulate gene availability and expression patterns; and epilepsy and other neurologic, metabolic, and myopathic abnormalities associated with mitochondrial dysfunction. Some of these findings overlap those presented within the present paper, although we have used alternative methods for phenotype clustering based on our earlier work.

Previously, we reported associations between autism and epilepsy comorbidities in monogenic (single-gene) ID with trends in functional gene enrichment, suggesting these behavioral/neurological phenotypes represent etiological divergence at the molecular level in at least some forms of ID (2). Here we show that additional secondary clinical features are also prominent, such as multiple congenital anomalies (MCA), neurodegeneration, brain atrophy, and motor disorders like upper motor neuron disease (UMND), all of which co-vary to greater or lesser degrees. Because of the prominence of these secondary clinical features, we have elected to extend similar work as Kochinke et al. (3) to perform in depth investigation into functional and modular enrichment in association with these clinical features, in the hopes that in using a more general approach across an array of different disorders we may identify previously unseen genotype-phenotype associations. It is our aim that this approach may allow us to group IDs into subtypes according to these gene-phenotype relationships, which may afford better understanding of their inherent biologies, as well as provide prognostic powers and potential cross-application of useful treatment paradigms.

In this study, we report multiple unique gene clusters with specific functional enrichment patterns that coincide with distinctive clinical phenotypes, indicating ID genes exhibit broad associations with observable phenotype.

## Methods

### Gene-phenotype curation

Our gene-ID dataset was curated as described in Casanova et al. (2). To summarize the curation process, a comprehensive list of different forms of ID with known molecular origins was accessed from the Mendelian Inheritance in Man (MIM) database (4). By selecting conditions with ID, we were able to estimate genetic penetrance for the autism and epilepsy phenotypes according to rates of comorbidity. Keywords for initial accession included: “intellectual disability,” “mental retardation,” “mentally retarded,” “global developmental delay,” “severe developmental delay,” and “profound developmental delay.” Any rare conditions not accessed by these call words were not included in the study for the sake of consistency. In addition, conditions were removed if they fulfilled any of the following criteria: (1) the ID was variably expressed and not considered a primary feature; (2) onset of ID was later than 3 years of age; (3) the condition was often lethal in infancy or early childhood; (4) the condition was considered genetically complex (e.g., deletion/duplication syndromes), with the exception of chromosome 2p16.3 deletion syndrome, which contains only the *NRXN1* gene; (5) autism was a defining symptom for diagnosis, as in the case of certain “susceptibility” genes; (6) the condition had <2 reported cases; (7) the condition was a chromosomal instability syndrome, leading to an accumulation of different mutations; and (8) the condition was demarcated by a “?” indicating an unconfirmed or potentially spurious mapping.

The larger group was then subdivided according to comorbidities with autism and epilepsy and their frequencies, which were verified both through MIM and the larger literature (see Additional File 2, Tables 2–4 tabs). For this study, only conditions with high autism and/or epilepsy rates, or without either comorbidity, were retained.

In addition, all conditions that were not contained within MIM's Clinical Synopses were removed, resulting in a final dataset of 212 different conditions. The autism group with/without epilepsy (referred to here as the “autism group”) contained 59 unique conditions; ID with epilepsy but without autism (referred to as the “epilepsy group”) was composed of 83 unique conditions; and ID without autism or epilepsy (ID group) was composed of 70 unique conditions. (see Additional File 2, “Table _1” tab for full list of IDs according to group and associated genes).

Comorbidity frequencies between ID and autism/epilepsy were obtained from the literature and described in detail in Casanova et al. (2). (In addition, see Additional File 2, Tables 2–4 for citation information). A high cut-off for inclusion within both the autism and epilepsy groups was ≥20% for all conditions for the sake of relative homogeneity. Only conditions without any indications of autism or epilepsy comorbidities, including the exclusion of single case examples, were placed within the ID group. This curation process culminated in three groups of conditions with very distinctive clinical and genetic profiles, as will be discussed in the Results section.

All conditions were annotated using the MIM's Clinical Synopses (12/15/2016), which represent common clinical features of a disorder and are organized anatomically. According to Amberger et al. (5), features included within Clinical Synopses:

…* are taken from the literature and incorporated into the synopsis using a semi-controlled vocabulary. Many features include modifiers and additional terminology specific to medical subspecialties that are helpful for delineating overlapping disorders and distinguishing characteristic features*. ***Among genetically heterogeneous disorders, care is taken to includeonly those features that are present in patients with mutations in the same causative gene****[our emphasis]*.

Conditions were annotated according to the presence of congenital anomalies in the following organs/tissues: the facial suite (face, eyes, ears, nose, mouth, dentition, neck); the cranial suite (cranial volume, synostoses, other cranial malformations, e.g., bitemporal narrowing); hands and feet; the limbs; the viscera and genitals (changes to the latter not otherwise due to peripubertal hypogonadism, etc.); hair and skin; and the brain [partial/complete agenesis of the corpus callosum and malformations of cortical development (MCD), the limbic system, the midbrain, and the brainstem, all visible via MRI]. Complex (CFD) and simple facial dysmorphia (SFD) were annotated according to the number of facial regions affected, rather than according to the number of specific dysmorphisms associated with a given condition. Tissue regions include overall facial shape; the nose; the exterior of the mouth; the interior mouth such as tongue, dentition, and jaw shape; the form of the eyes; the midface (cheeks); and the ears. CFD was defined according to three or more malformations in distinct tissue regions, while SFD was defined as 1–2.

Phenotype interactions were analyzed across all congenital anomalies. Following analysis (see Results), CFD was selected as a defining secondary clinical feature for further genetic study, due both to clinical prominence and predictive ability in the presence of MCA syndromes. SFD were also selected as a secondary feature of interest for the sake of contrast, although were generally not predictive of MCA syndromes.

Conditions were also annotated for the presence of: neurodegeneration (confirmed according to literature search); brain atrophy; symptoms indicative of UMND, such as spasticity and hyperreflexia; and the presence of symptoms indicating the co-occurrence of 2 or more distinct movement disorders [UMND, lower motor neuron disease [LMND], disorders of the cerebellum, and disorders of the basal ganglia]. Because brain atrophy and motor disorders were positively associated with neurodegeneration (see Results), all of the above clinical features were collapsed into a single category, “neurodegenerative-like features (NLF),” for the purposes of further genetic study. CFD and NLF phenotypes were further substantiated using the Human Phenotype Ontology (HPO) database, and, when that was insufficient, the general literature in order to ensure reliability of MIM's Clinical Synopsis results for each of the conditions studied (6).

**Figure 1:**
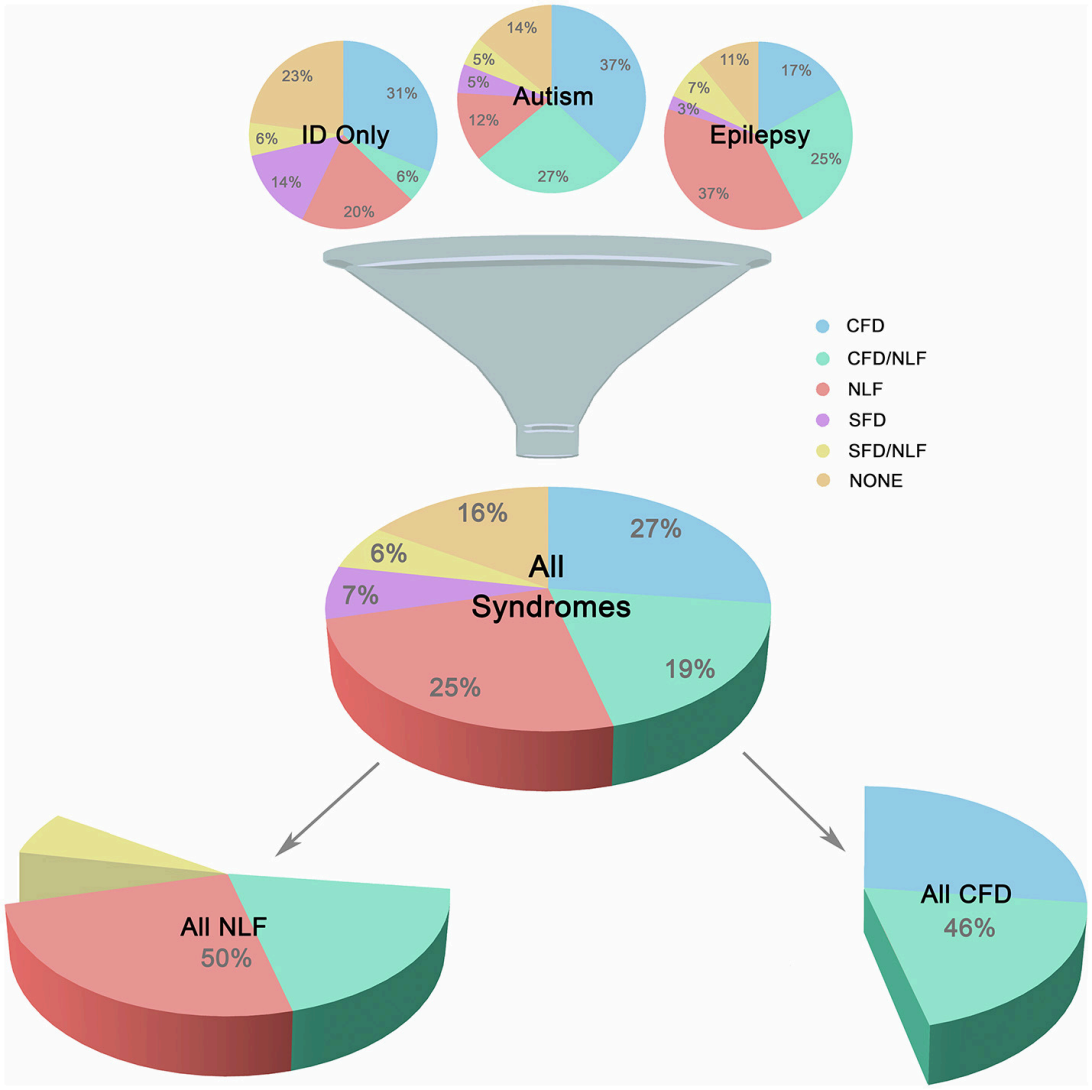
Major Subgroups. Illustration showing breakdown of the three main groups (autism, epilepsy, and ID only) into smaller clinically-related subgroups. Subgroups were defined according to the presence of complex facial dysmorphia (CFD), neurodegenerative-like features (NLF), simple facial dysmorphia (SFD), or a lack of these same features (i.e., “none”). As shown in the lower portion of the image, CFD, and NLF overlapped ~20% of the time, although analyses indicate no clear relationship between the two sets of features, suggesting possible genetic pleiotropy when comorbid.

In order to study the association of the above clinical phenotypes with autism, epilepsy, and ID groups, conditions were subdivided according to the overlapping clinical phenotypes presented in Figure 1. This resulted in 18 unique gene sets, composed of 216 genes representing 212 different forms of monogenic ID (Additional File 2, “Table _1” tab).

### Extended gene interaction network

The GeneMANIA gene interaction database [genemania.org; (7)] was queried to discover additional known interactions for all 216 curated seed genes (Additional File 3, Tables _15–17 tabs). The database provides a report containing several different interaction types including physical, genetic, pathway, predicted, co-localization, co-expression, and shared protein domains. All interactions were obtained from the “networks.data” link, but for the purpose of this study only genes with physical, genetic, or co-expression interactions were included in the finalized network (Additional File 3, Tables 16–17 tabs).

Visualization and analysis of the network was conducted via Cytoscape (8). The “Network Analysis” Cytoscape app was used to determine topological parameters including node degree distribution fit to the power law, centrality, average connectivity, and clustering co-efficient. The clusterMaker MCL algorithm (granularity 1.2) in the Cytoscape clusterMaker app was used to identify highly connected gene clusters in the extended network (http://www.cgl.ucsf.edu/cytoscape/cluster/clusterMaker.shtml). The clusterMaker algorithm is a plugin that partitions clusters into “meta nodes,” allowing interactive exploration of putative associations.

Kochinke et al. (3) reported that nearly half of all ID genes physically interact with one another, with more than a third forming a single large interactive network. Therefore, we tested if phenotype labels, assigned at the gene curation stage, and their extended interactions were non-randomly enriched in MCL gene clusters using the Fisher's Exact Test (*p* < 0.001). Label enrichment was performed on the observed clusters. Fisher's test addresses the potential relationship of these clusters without the need to randomize genes between clusters or create random networks for label enrichment analysis (see Additional File 1).

In addition, because there is a portion of genes within the autism gene group that are not currently contained within the syndromic category of the SFARI gene database and may therefore be suspect, we have also assessed nonrandom clustering of syndromic SFARI seed genes to illustrate that similar clustering still occurs with more stringent exclusion criteria. Our approach was identical as in the full network analysis, with the exception that only seed genes contained within the syndromic SFARI category were used (9).

Enrichr was used for functional enrichment analysis of each phenotypically-driven subgroup gene list (*N* = 570 total genes) for these annotation categories: Gene Ontology (GO), KEGG/WikiPathways, TRANSFAC/JASPAR Position Weight Matrix (PWM), MGI Mammalian Phenotype (MP), and Human Phenotype Ontology (HPO) (10). The Enrichr platform provides adjusted *p*-values using the Enrichr list randomization method, which is based on the Fisher's Exact test as well as *Z*-scores and combined scores for each annotation and is fully explained in Chen et al. (11). As a guide of possible collective gene function for each gene list, we used an adjusted significance *p*-value threshold of *p* < 0.05.

### Statistical analyses

For phenotype analyses, between- and within-group comparisons were performed using two-sample proportion Chi-square tests with a false discovery rate *p*-value adjustment (R pairwise.prop.test). Odds ratios with sample size adjustments (12) were computed to examine associations amongst different congenital anomalies, as well as associations within NLF, the latter without sample size adjustment.

## Results

### Clinical features common in monogenic intellectual disability

Congenital anomalies are prominent features within monogenic forms of ID. In this study, the most common congenital anomaly reported was CFD, occurring in almost half of the conditions studied. Less frequent though still prominent congenital anomalies included (in order of frequency from most to least): microcephaly; organ malformations; brain malformations (visible via MRI); craniosynostoses and other cranial malformations; hand and foot malformations; skin and hair disturbances; SFD; macrocephaly; and limb malformations.

Not only was CFD the most common dysmorphism, it is also strongly associated with other types of dysmorphia (*z* = 0.7813–7.1947, *p* < 0.001–0.014; *OR* = 2.19–13.461; *OR CI* = 1.261–6.266, 3.805–28.717), with the exception of specific brain malformations (*z* = 0.328–2.230, *p* = 0.055–0.814; *OR* = 1.159–1.744; *95% CI* = 0.479–1.108, 2.654-4.892; see Additional File 3, “Table _11” tab for full results). One primary exception was the strong relationship between complete/partial agenesis of the corpus callosum (ACC) and CFD, suggesting significant etiological links (*z* = 2.993, *p* = 0.009; *OR* = 4.465; *95% CI* = *95% CI* = 1.762, 15.117). Microcephaly was also only very weakly predictive of MCA (aside from brain and cranium; *z* = 1.113–2.781, *p* = 0.014–0.369; *OR* = 1.393–2.19; *95% CI* = 0.734–1.261, 2.498–3.948), and therefore facial dysmorphia were annotated separately from deviations in cranial volume in this study, despite the clinical tradition of grouping all craniofacial malformations together.

Neurodegeneration was also common occurring in ~20% of ID and was an extremely strong predictive factor for the presence of brain atrophy and various movement disorders, especially UMND (*z* = 5.110, *p* < 0.001; *OR* = 8.61; *95% CI* = 3.77, 19.68; see Additional File 3, “Table _13” tab). Another ~30% of conditions displayed either brain atrophy, UMND, or multiple movement disorders (MMD; or some combination thereof) but are not currently recognized as classical neurodegenerative disorders. However, because of their strong interrelationship suggesting linked etiologies, neurodegeneration, brain atrophy, UMND, and MMD were combined under a single heading, “neurodegenerative-like features” or “NLF,” for the purposes of this study (*z* = 4.69–8.73, *p* < 0.001; *OR* = 4.64–56.53; *95% CI* = 2.44–22.86, 8.82–139.82). NLF occurred in 50% of the conditions studied, overlapping CFD ~19% of the time. Despite this large overlap, in the majority of cases these features did not co-occur and, overall, exhibited no statistically significant relationship with one another (*p* = 0.515; *OR* = 0.834; *95% CI* = 0.483, 1.440). This suggests that while these phenotypes may co-occur in a large minority of these conditions, they are nevertheless unique symptom clusters and may instead reflect genetic pleiotropy (i.e., a single gene influences 2 or more unrelated traits) when comorbid (see Figure 1).

Previous results by Casanova et al. (2), utilizing a near-identical dataset, indicate a divergence in functional gene enrichment in ID according to autism and epilepsy comorbidities. Here we report additional clinical phenotype enrichment that varies according to these behavioral/neurological comorbidities. Namely, the autism group was significantly enriched for the presence of CFD (61% vs. 37–40%), suggesting many rare autism syndromes may be dysplastic in nature (χ^2^ = 5.42-6.38, *p* = 0.03) (13–15) (see Additional File 3, “Table _12” tab). Meanwhile, the epilepsy group was similarly enriched for NLF (68% vs. 31–39%), indicating some form of cell stress may be involved in these IDs (χ^2^ = 11.18–19.63, *p* < 0.001) (16, 17). There are additional clinical phenotypes that vary according to group, such as enrichment of neocortical malformations (identified by MRI; *z* = 4.4566, *p* < 0.001, *OR* = 6.4289; *95% CI* = 2.836, 14.573) and microcephaly (*z* = 2.8656, *p* = 0.011, *OR* = 2.2778; *95% CI* = 1.297–4.000) in the epilepsy group. (For full results, see Additional File 3, “Table _14” tab).

### ID genes cluster according to phenotype

Using a list of 216 seed genes divided according to our phenotypes of interest, we have identified an additional 354 interacting genes using the GeneMANIA gene interaction database (genemania.org). This resulted in the formation of 17 unique gene sets composed of a total of 1,195 genes upon which to perform gene module detection according to all protein-protein interaction (PPI), genetic interaction, and mRNA co-expression connections. One of the autism subgroups failed to show any significant intracluster interactions and therefore was not included in the cluster and functional enrichment analyses.

**Figure 2:**
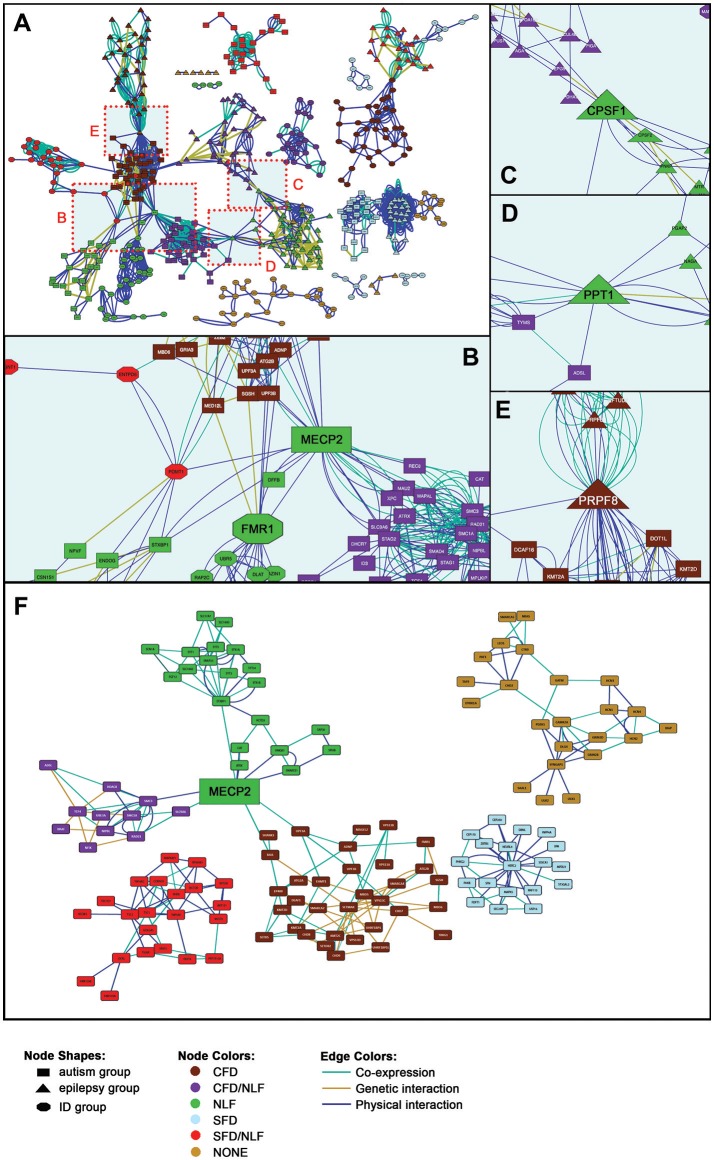
Gene Interaction Network. **(A)** Full gene interaction network. (See Supplementary Figure 1 for detailed gene network). **(B)** Autism-linked *MECP2* and *FMR1* hubs. **(C)** Epilepsy-linked *CPSF1* hub. **(D)** Epilepsy- and autism-linked *PPT1* hub. **(E)** Epilepsy- and autism-linked *PRPF8* hub. **(F)** Syndromic SFARI gene interaction network. (See Supplementary Figure 2, for detailed gene network).

As can be seen in Figure 2A, the seed genes plus PPI, genetic interacting, and co-expression loci form 17 sets of relatively non-overlapping gene clusters, constituting tight interaction/coexpression networks. Thirteen of the 17 gene sets form particularly tight clusters and are interconnected via specific hub nodes (Figures 2B–E). (For detailed views of the full cluster network, see Additional File 1, Figure 1) Overall network degree distribution modestly fits the power law distribution (*r* = 0.776), indicating the network trends toward scale free behavior (i.e., clustering is non-random and potentially reflects a real gene interaction networks). Other topological parameters of interest include: clustering coefficient = 0.342; centralization = 0.034; and average connectivity = 5.287. SFARI-only syndromic genes likewise formed similar non-random clusters (*r* = 0.687), indicating the robustness of the autism results overall (Figure 2F). Overall, these results indicate that our genes of interest form nonrandom interaction clusters that naturally fall within clusters according to the phenotypes of interest (CFD, NLF, SFD, etc.), suggesting that these phenotypes are strong predictors to which gene cluster, if any, a given gene belongs.

Within the main network, more than half of the gene sets are interconnected via 10 hub nodes (genes onto which the major clusters converge; Figures 2B–E). The Rett syndrome-associated gene, *MECP2*, for instance, forms a hub connecting half of the autism-related gene sets, particularly those with secondary clinical features of CFD, combined CFD/NLF, and pure NLF, as well as connecting one of the ID group clusters (Figure 2B). In addition, *MECP2* remains an important hub node in the SFARI-only syndromic network, continuing to link CFD, CFD/NLF, and NLF autism subgroups. MECP2's nature as a semi-ubiquitous repressor of long genes, which typifies many neural genes, places it in a key position to regulate development of the central nervous system and thus to potentially interact with many of the genes presented here (18).

Likewise, the Fragile X syndrome-associated gene, *FMR1*, forms a major hub connecting the same clusters as *MECP2* within the main network, although this result is not maintained within the abbreviated SFARI network (Figures 2B, F). Interestingly, like MECP2, there is some evidence to suggest that FMRP specifically targets gene products translated from long genes, suggesting MECP2 and FMRP may regulate different points along many of the same pathways (19, 20).

Two other major hubs in the main network are involved in mRNA processing: *CPSF1*, which is involved in 3′ processing of mRNA, and *PRPF8*, which acts as a scaffold for spliceosomal complexes and snRNA. As shown in Figure 2C, *CPSF1* connects two epilepsy modules with features of NLF; meanwhile, *PRPF8* (Figure 2E) connects epilepsy/CFD (EPI/CFD) with autism/CFD (AUT/CFD). As we shall see in the following section, a number of the epilepsy clusters are enriched for mRNA processing. Interestingly, PRPF8 is also essential for sister chromatid cohesion, making it therefore surprising that it forms a hub with AUT/CFD rather than AUT/CFD/NLF, as we shall see in the following section (21).

Finally, the hub, *PPT1*, whose mutation is responsible for the neurodegenerative and lethal condition, Neuronal Ceroid Lipofuscinosis 1, links the EPI/NLF and AUT/CFD/NLF modules (Figure 2D). As a glycoprotein involved in catabolism of lipid-modified proteins and a regulator of heat shock proteins, its loss results in excessive generation of reactive oxygen species (ROS) (22, 23). *PPT1*'s role as a hub linking EPI/NLF and AUT/CFD/NLF can potentially be viewed in light of the roles chronic ROS play in the synaptic impairment that ultimately leads to a host of neurodegenerative disorders (24).

### Functional enrichment trends in gene subgroups

The genes sets in some phenotype subgroups showed little obvious trends in functional enrichment, such as EPI/SFD/NLF and ID/SFD. This may be a reflection of etiological diversity in these respective modules and/or the inadequacy of current platforms in estimating disparate functional relationships.

**Figure 3:**
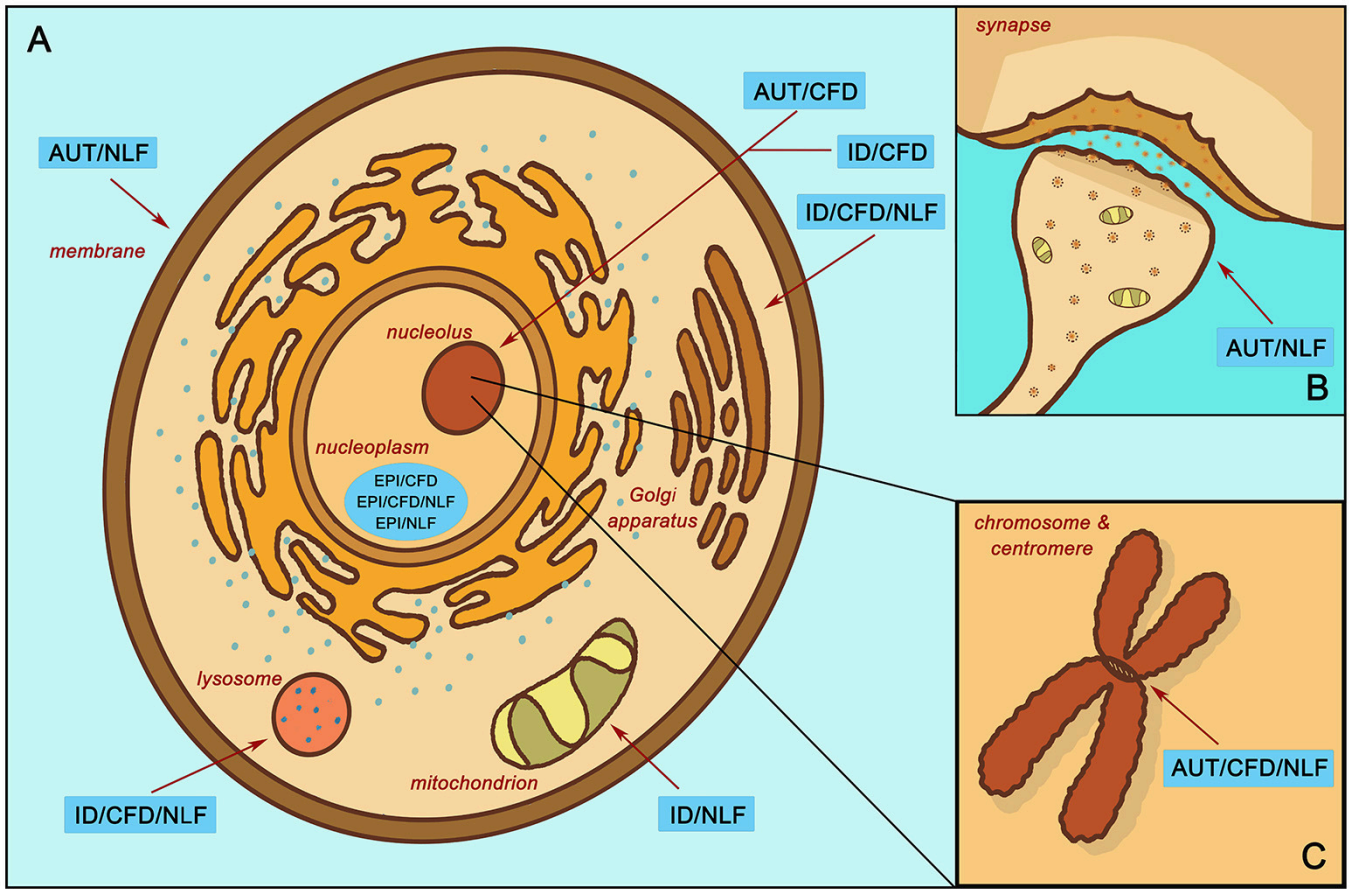
Enrichment localization according to subgroup. **(A)** Localized enrichment according to subgroups within the main body of the cell. **(B)** Subgroup enrichment within the synapse. **(C)** Subgroup enrichment at the chromosome and centromere. Intellectual disability with autism and with/without epilepsy (AUT); intellectual disability with epilepsy and without autism (EPI); intellectual disability without autism or epilepsy (ID); complex facial dysmorphia (CFD); neurodegenerative-like features (NLF).

Other groups, however, appeared to show distinctive functional trends, particularly those associated with CFD. For instance, the AUT/CFD gene subgroup is strongly enriched for processes relating to *chromatin modification* (*z* = −2.40, *p* < 0.001), *histone modification* (*z* = −2.39, *p* < 0.001), *methylation* (*z* = −2.45, *p* = 0.007), *transcription factor binding* (*z* = −2.16, *p* = 0.026), and is localized to the nucleus (*nucleolus*; *z* = −2.21, *p* = 0.002; Figure 3A). All of these enrichments strongly implicate AUT/CFD genes in the regulation of gene expression and, ultimately, organ and tissue development.

More than a third of AUT/CFD genes are also transcriptional targets for Wilms tumor suppressor 1 (Wt1), a transcription factor that helps regulate cell development and survival (*z* = −1.62, *p* = 0.036). In addition, almost half of AUT/CFD genes are transcriptional targets of Lef1, a positive regulator of the canonical Wnt pathway, which is itself a foundational network involved in organ and tissue morphogenesis (*z* = −1.48, *p* = 0.036) (25).

In contrast, the EPI/CFD gene subgroup, though likewise relegated to the *nucleoplasm* (*z* = −2.16, *p* < 0.001) and involved in *histone modification* (*z* = −2.39, *p* < 0.001), is also enriched for processes involved in *mRNA processing* (*z* = −2.37, *p* = 0.003) and the *spliceosomal complex* (*z* = −2.15, *p* < 0.001). Similarly, EPI/CFD/NLF was enriched for *RNA polyadenylation* (*z* = −2.66, *p* < = 0.003). Many of these functions concern post-transcriptional stages of gene expression regulation, while enrichments associated with AUT/CFD involve regulation of transcription itself. ID/CFD meanwhile is enriched in *kinase binding* (*z* = −2.55, *p* = 0.011) and *chromatin binding* (*z* = −2.45, *p* = 0.031), while ID/CFD/NLF is enriched for *protein glycosylation* (*z* = −2.34, *p* < 0.001) and is localized to the *Golgi membrane* (*z* = −2.29, *p* > 0.001) and the *lysosome* (*z* = −2.31, *p* > 0.001). All CFD enrichments strongly implicate the role of gene expression regulators in the pathophysiology of complex facial dysmorphia.

When comparing the two autism CFD subgroups to one another, we found that both AUT/CFD and AUT/CFD/NLF are involved in *chromatin binding* (*z* = −2.47, *p* < 0.001). However, AUT/CFD/NLF is also strongly enriched for processes involving the *mitotic cell cycle* (*z* = −2.30, *p* < 0.001) and *sister chromatid cohesion* (*z* = −2.67, *p* < 0.001), which is entirely missing from the AUT/CFD gene subgroup (Figure 3B).

In contrast to its CFD counterparts, ID/NLF was enriched in *hydrogen ion membrane transporter activity* (*z* = −2.34, *p* = 0.003) and was involved in the *respiratory chain* (*z* = −2.59, *p* < 0.001) within mitochondria. In addition, it displayed pathway enrichment in relation to *Parkinson's disease* (*z* = −1.77, *p* < 0.001), *Huntington's disease* (*z* = −1.85, *p* = 0.002), and *Alzheimer's disease* (*z* = −1.72, *p* = 0.015). The EPI/NLF gene subgroup, in contrast, was enriched for a variety of terms, such as *myelin sheath* (*z* = −2.89, *p* < 0.001), *mRNA polyadenylation* (*p* = 0.007, *z* = −2.71), *carboxylic acid biosynthetic process* (*z* = −2.35, *p* = 0.007), and *protein folding* (*z* = −2.31, *p* = 0.007), suggesting that despite strong intracluster connectivity, the etiology of the EPI/NLF subgroup is comparatively diverse. Meanwhile, AUT/NLF was modestly enriched for *membrane depolarization* (*z* = −2.26, *p* = 0.005), *regulation of postsynaptic membrane potential* (*z* = −2.09, *p* = 0.009), and *regulation of synaptic plasticity* (*z* = −2.15, *p* = 0.027) (Figure 3C). This indicates that disturbances to synaptic proteins in autism could be related to symptoms of NLF, an idea that may be worthy of further exploration in relation to autistic regression given the role of synaptic impairment in the etiologies of many neurodegenerative disorders (26). Interestingly, recent research indicates that autistic individuals with gene disrupting mutations in postsynaptic density genes are more likely to experience autistic regression than individuals with mutations in genes of other functional classes (27) (see Additional File 4, “Table _19” tab for more extensive enrichment results by subgroup).

## Discussion

The present study provides evidence of genotype-phenotype correlations throughout multiple ID subsets. In particular, the presence of autism (with or without epilepsy), epilepsy (without autism), CFD, and NLF appear to be general predictors of associated gene function. The AUT/CFD gene subgroup, for instance, is linked with genes localized to the nucleus. These genes are involved in chromatin modifications; histone modifications; methylation; transcription factor binding; and are key in regulating embryonic development. In contrast, gene products of the AUT/CFD/NLF subgroup are likewise localized to the nucleus and involved in chromatin binding, but are typically involved in regulation of the cell cycle and sister chromatid segregation. Nuclear localization appears to be a strong risk factor in the developments of both autism and CFD in these subgroups; however, cell cycle involvement may provide an additional risk for NLF as we see in many classical neurodegenerative diseases (28).

Nuclear localization, in general, seems to be a strong predictive factor for the presence of CFD and, more weakly, SFD, although specific functional enrichments vary with the presence of autism, epilepsy, and NLF accordingly. AUT/CFD, EPI/CFD, and ID/CFD all tend to be localized to the nucleus and are at least modestly enriched in processes relating to chromatin binding and modifications. The important roles these gene expression regulators play in organogenesis likely underlie their associations with complex facial dysmorphia and other physical features.

We have also identified a number of major hubs within the clusters analysis, linking otherwise non-overlapping gene sets. Although functional relevance of some of the hubs is currently uncertain, several of the autism hubs are already major foci within the current literature. *MECP2*, the primary gene responsible for Rett syndrome, and *FMR1*, the gene associated with Fragile X syndrome, have both received considerable attention and *FMR1* in particular has been previously identified as a major pathway of interest in the pathophysiology of autism (29–31). From the clinical perspective, both Rett and Fragile X syndromes share strong associations with autism. Most girls with Rett's present with transient autistic features at a characteristic stage within the prolonged regressive period. Meanwhile, approximately half of individuals with Fragile X present with an enduring autism phenotype (32). Despite their unique clinical phenotypes, our data indicate that both *MECP2* and *FMR1* form foundational pathways underlying autism risk and may overlap in part due to their roles as major regulators of neuronal gene expression and protein translation.

Finally, we have shown that specific secondary clinical phenotypes exhibit strong association with ID according to comorbidities with autism and epilepsy. For instance, the high rates of CFD and MCA in rare autism syndromes are strongly suggestive of a common biology despite genotypic variation. Despite the dearth of obvious brain malformations reported in our autism dataset, the high prevalence of microscopic dysplastic foci in idiopathic autism tends to validate this point (13, 15, 33, 34).

Our results have also shown that close to half of the conditions studied here exhibit features reminiscent of neurodegeneration, although only about a fifth are officially recognized as “neurodegenerative disorders.” The occurrence of NLF is particularly prominent in the epilepsy group, although functional enrichment of the ID/NLF subgroup is more aligned with processes of classic neurodegeneration. However, these data suggest that: (1) postmortem analysis of neurodegeneration may be understudied in some of these conditions, and/or (2) proteopathies with obvious inclusions may comprise only a subset of a broader range of neurodegenerative-like disorders, which have subtler, more complex etiologies with progressions that differ from the typical dementias that occur in later life. In support of this, Sarnat and Flores-Sarnat (35) have recently addressed such concepts within the context of “infantile tauopathies,” such as tuberous sclerosis and focal cortical dysplasia 2. At present, recognized infantile proteopathies include only those conditions resultant from MTOR overexpression, a known mechanism of neurodegeneration (36). However, given the range of inclusion bodies associated with adult forms of neurodegeneration and senile dementias, the list of infantile proteopathies is likely to expand in future and may eventually be recognized as a major cause of some developmental and intellectual disabilities (35, 37).

### Current limitations and future research

Given the nature of the MIM database, whose purpose is intended to summarize genetic and syndromic disease states, research procedures have varied across individual studies that compose the MIM. The state of the MIM is also potentially incomplete, leading to gaps in our dataset. For these reasons, our results must be extrapolated cautiously, requiring further investigations at the clinical and molecular levels. However, although the MIM data may be incomplete, we feel the current dataset provides an excellent overview of the major gene-phenotype trends that are currently available for data mining. In addition, in order to limit the extent of Type I errors, we have elected to study clinical phenotypes whose medical evaluations are standardized across health fields, ensuring that the clinical data reported here may be relatively reliable (38, 39).

One major exception to this is the field of autism diagnostics, which has changed significantly over the past 25 years. A majority (59%) of seed genes used in this analysis is included within the syndromic category of the SFARI Gene Database, supporting their diagnostic reliability in this study. While we are unable to directly address diagnostic reliability of the remainder of autism genes, we instead assessed robustness of non-random clustering of this subset of syndromic SFARI genes, which like the larger autism gene group exhibited similar clustering. This supports our general findings as well as potential risk status of non-SFARI genes included in this study.

Another limitation of the study is the question of its applicability to a broader range of conditions. The study of severely affected individuals with rare genetic syndromes is a common approach to investigating human illness in order to better understand complex conditions. However, such assumptions are based on symptom similarity rather than biological evidence. As such, our results may not apply to forms of ID, autism, and epilepsy that lack strong genetic roots. However, recent work by Rossi et al. (40) suggest that even those patients with autism but without obvious syndromes often harbor potentially deleterious variants in many of the same genes studied here. Further lines of research will continue to address potential cross-applicability of the data presented here. In the meantime, we believe the subgroups we've described can provide a platform for the further elucidation of common denominator pathways and the regulatory networks underlying these complex conditions, leading to the subtyping of disorders.

## Conclusions

The present study provides strong evidence that ID-associated phenotypes cluster according to related gene function. Specifically, gene modules form according to autism, epilepsy, CFD, and NLF comorbidities. Future research will help to delineate these subgroups in greater detail, as well as determine whether additional genotype-phenotype correlations exist in these and related datasets.

## Authors note

Emily L. Casanova, Ph.D., is a postdoctoral fellow in Biomedical Sciences at the University of South Carolina Greenville Medical School with training in developmental and molecular biology and a focus on neurodevelopmental disorders. Zachary Gerstner is a graduate student in Genetics and Biochemistry at Clemson University with training in microbiology, genetics, and computer science. Julia L. Sharp, Ph.D., is an associate professor and director of the Graybill Statistical Laboratory at Colorado State University. She is an applied statistician with expertise in experimental design and mixed models. Manuel F. Casanova, MD, is the SmartState Endowed Chair in Childhood Neurotherapeutics for the University of South Carolina and the Greenville Health System. His clinical and research focus concerns neurodevelopmental disorders with an emphasis on autism. F. Alex Feltus, Ph.D., is an associate professor in the Department of Genetics and Biochemistry at Clemson University with 23 years of broad experience in bioinformatics, systems genetics, and genomics.

## Availability of data and material

All data generated and analyzed during this study are included in this published article and its Supplementary Information files. Full statistical results are also available, as well as additional results that support the main text, such as Additional File 4, “ID_Comparison” and “No_Cases” tabs.

## Author contributions

EC and FF conceived the study. EC curated the phenotypic data and JS performed statistical analyses on that data. ZG and FF performed cluster and enrichment analyses and associated statistics. MC provided expertise on autism, intellectual disability, and epilepsy and was integral in helping design the study as well as interpret results. All authors contributed substantially to the drafts and have read and approved the final manuscript.

### Conflict of interest statement

The authors declare that the research was conducted in the absence of any commercial or financial relationships that could be construed as a potential conflict of interest.

## Abbreviations

ID: Intellectual disability
CFD: complex facial dysmorphisms
SFD: simple facial dysmorphisms
NLF: neurodegenerative-like features
MCA: multiple congenital anomalies
UMND: upper motor neuron disease
MMD: multiple movement disorders
PPI: protein-protein interaction.
