# Supplementary figures and images for: Widespread Genotype-Phenotype Correlations in Intellectual Disability
Source: Front Psychiatry. 2018 Oct 29;9:535. doi: 10.3389/fpsyt.2018.00535 (PMC6217001; doi:10.3389/fpsyt.2018.00535)

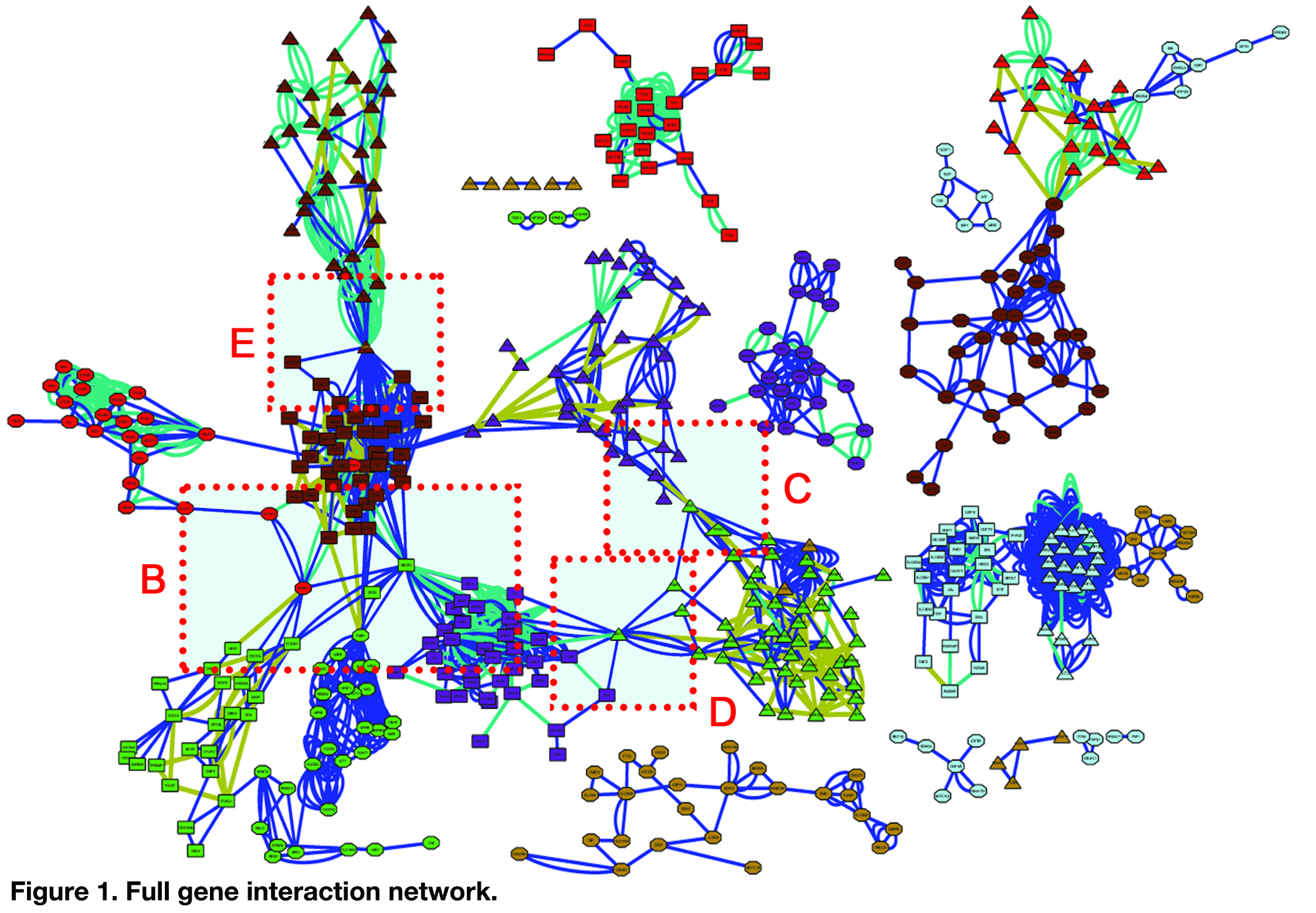

Supplement: Supplementary file 4 [file Image_1.tif]

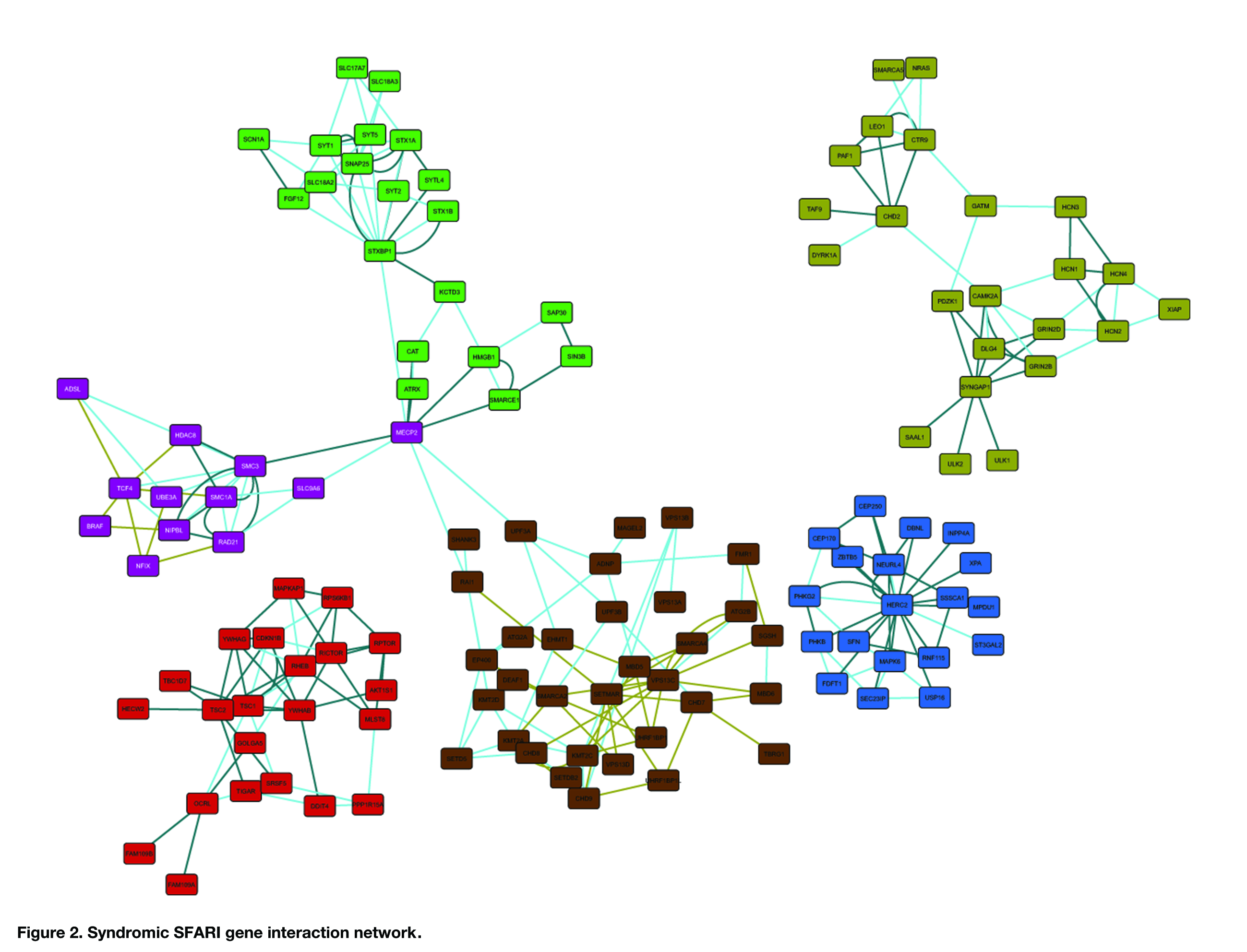

Supplement: Supplementary file 5 [file Image_2.tif]
